# Prevalence and correlates of depression and anxiety among patients with HIV on-follow up at Alert Hospital, Addis Ababa, Ethiopia

**DOI:** 10.1186/s12888-016-1037-9

**Published:** 2016-11-02

**Authors:** Getachew Tesfaw, Getinet Ayano, Tadesse Awoke, Dawit Assefa, Zelalem Birhanu, Getenet Miheretie, Genet Abebe

**Affiliations:** 1 Department of psychiatry, Felege Hiwot Referral hospital, Bahirdar University, Bahirdar, Ethiopia; 2 Research and training department, Amanuel mental specialized hospital, Addis Ababa, Ethiopia; 3 Institute of Public Health, College of Medicine and Health Sciences, University of Gondar, Gondar, Ethiopia; 4 Department of psychiatry, College of Medicine and Health Sciences, University of Gondar, Gondar, Ethiopia; 5 Department of non communicable diseases, Addis Ababa Health office, Addis Ababa, Ethiopia

**Keywords:** Depression, Anxiety, HIV/AIDS

## Abstract

**Background:**

Depression and anxiety disorders are common among people living with Human Immunodeficiency Virus than the non-infected individuals. The co-existence of these disorders are associated with barriers to treatment and worsening medical outcomes, including treatment resistance, increased risk for suicide, greater chance for recurrence and utilization of medical resources and/or increase morbidity and mortality. Therefore, assessing depression and anxiety among HIV patients has a pivotal role for further interventions.

**Methods:**

Institution based cross-sectional study was conducted at ALERT hospital May, 2015. Data were collected using a pretested, structured and standardized questionnaire. Systematic sampling technique was used to select the study participants. Binary logistic regression analysis was used to identify associated factors. Odds ratio with 95 % CI was computed to assess the strength of associations.

**Results:**

The prevalence of co-morbid depression and anxiety among HIV patients was 24.5 % and prevalence of depression and anxiety among HIV patients was 41.2 % (172) and 32.4 % (135) respectively. Multivariate analysis showed that individual who had perceived HIV stigma (AOR = 3.60, 95 % CI (2.23, 5.80), poor social support (AOR = 2.02, 95 % CI (1.25, 3.27), HIV stage III (AOR = 2.80, 95 % CI (1.50, 5.21) and poor medication adherence (AOR = 1.61, 95 % CI (1.02, 2.55) were significantly associated with depression. Being female (AOR = 3.13, 95 % CI (1.80, 5.44), being divorced (AOR = 2.51, 95 % CI (1.26, 5.00), having co morbid TB (AOR = 2.74, 95 % CI (1.37, 5.47) and perceived HIV stigma (AOR = 4.00, 95 % CI (2.40, 6.69) were also significantly associated with anxiety.

**Conclusion:**

Prevalence of depression and anxiety was high. Having perceived HIV stigma, HIV Stage III, poor social support and poor medication adherence were associated with depression. Whereas being female, being divorced and having co morbid TB and perceived HIV stigma were associated with anxiety. Ministry of health should give training on how to screen anxiety and depression among HIV patients and should develop guidelines to screen and treat depression and anxiety among HIV patients.

## Background

Currently, global statistics shows that an estimated 35 million people were living with HIV/AIDS in 2013, of which 24.7 million are living in Sub-Saharan Africa and 1.6 million people died related to AIDS [1]. In 2012 estimation, 9.5 million people in developing countries were receiving HIV treatment [2]. National prevalence in Ethiopia was 1.3 % and 137 people receive ART in all ages [1].

There is a significant psychological impact imposed among HIV/AIDS patients. People with HIV often suffer from depression and anxiety as they adjust to the impact of the diagnosis of being infected and face the difficulties like shortened life expectancy, complicated therapeutic regimens, stigmatization, and loss of social and family support. HIV infection can also be associated with high risk of suicide [3–5].

Depression is a common mental disorder that presents with depressed mood, loss of interest or pleasure, decreased energy, feelings of guilt or low self-worth, disturbed sleep or appetite, and poor concentration [6]. Approximately, 350 million people are currently living with depression [6]. It is the fourth leading cause of disability worldwide and it will become the second leading cause of disability by 2020 [7]. Its life time prevalence was estimated to be approximately 3 to 17 % [8].

In Ethiopia the survey done in nine regions reported, the prevalence of depressive episode was 9.1 %, which was associated with age, divorced, widowed, and alcohol drinking status and women are two times affected as compared to men with depression [9, 10].

Anxiety is a vague, subjective, non-specific feeling of uneasiness, apprehension, tension, (excessive nervousness) fears, and a sense of impending doom, irrational avoidance of objects or situations and anxiety attack [6]. It was associated with gender, age, and chronic diseases [11]. Women were affected nearly two times as compared to men by anxiety [8].

Depression and anxiety disorders are common in HIV infected individuals than general population [5]. Depression always coming with the symptom of anxiety and they have cause and effect relationship with chronic diseases [6, 12]. Depressive disorder was three times more common among HIV infected individuals and its lifetime prevalence estimate was 22–45 % [12, 13]. Depression among HIV patients leads to low adherence to medical treatment, an increase health cost disability and mortality [14]. HIV/AIDS infected individuals are more prone for depression and anxiety disorders which, in turn, increase stigmatization, decrease quality of life, increase mortality, reduce adherence and impair their immune function [15].

Risk factors associated with depression and anxiety among HIV infected patients were low income, unemployment, alcohol use, poor medication adherence, HIV/AIDS stage, perceived stigma and social support [16–18]. Even though the co-morbidity of depression and anxiety among HIV/AIDS patients is a major cause of morbidity and mortality in developing countries like Ethiopia, little attention is given for their diagnosis. Therefore, the finding of this study will link the gap in making necessary solution by assessing the prevalence and factors associated with depression and anxiety among people living with HIV/AIDS patients in the study area.

## Methods

### Study setting and design

Institution based cross-sectional study was conducted at ALERT hospital May, 2015,Addis Ababa, Ethiopia.

### Study population

The study population consisted of all adult HIV patients who were on follow up at ALERT hospital who were included in the sample. Those HIV patients who were critically ill were excluded from the study.

### Sampling procedures

Sample size was determined based on single population proportion formula using Epi-info version 7 with a 95 % CI, 5 % margin of error and taking prevalence of depression and anxiety 43.9 % [19] and 22.2 % [20] respectively. Assuming a 10 % non-response rate a total sample size of 417 HIV cases was required. Systematic sampling technique was used to select the study participants. Sampling interval was determined by dividing the total study population who had follow-up during four weeks data collection period by total sample size then the starting point was randomly selected.

#### Data collection

Data were collected using pretested interviewer administered questionnaire, which contains socio-demographic characteristics (age, education, occupation, marital status and others), Perceived HIV stigma (patients who scored greater than or equal to mean (≥18.66 or ≥5.54) on 11-item perceived HIV stigma scale), Social support (individuals who were scored greater than or equal to 9 (moderate and strong) on Oslo 3- item social support scale), Depression (depression was measured by using seven items of (depression sub scale) of Hospital Anxiety and Depression Scale (HADS)with cut-off points of greater than or equal to 8 scores) and Anxiety (anxiety was measured by using seven items of (anxiety sub scale) HADS with cut-off points of greater than or equal to 8 scores).

### Data processing and analyses

Data were analyzed using SPSS version 20. Description of means, frequencies, proportions and rates of the given data for each variable was calculated. Bivariate analysis was done to see the association of each independent variable with the outcome variable. Those variables having *p*-value less than 0.2 were entered into the multivariate logistic regression model to identify the effect of each independent variable with the outcome variables. A *p*-value of less than 0.05 was considered statistically significant, and adjusted odds ratio with 95 % CI was calculated to determine association.

### Ethical consideration

Ethical clearance was obtained from the Institutional Review Board of the University of Gondar and Amanuel Mental Specialized. A formal letter of permission was obtained from Amanuel mental specialized hospital and University of Gondar was submitted to AHRI/ALERT ethical review committee for getting ethical clearance to do on the site. Supportive letter was obtained from AHRI/ALERT. Written informed consent was obtained from each study participant after they were introduced to the purpose of the study and informed about their rights to interrupt the interview at any time. Participants were informed that the information collected for this research project were kept confidential and information about collected by this study will be stored in a file, without their name, but code number assigned to it. It will not be revealed to anyone except the principal investigator and during final dissemination of aggregate results on workshops, publications and the like. Any individual level clinical data will not be disseminated for any one and it will be kept locked with key. HIV/AIDS patients who were found to have depression and anxiety were referred for further investigations.

## Results

### Socio-economic and demographic characteristics

A total of 417 participants were included in the study which makes the response rate 100 %. Themean age of the respondents was 37.44 (± standard deviation = 10.07) years.

Among total participants, 251 (60.2 %) were female, 149 (35.7 %) were married,157 (37.7 %) were between the ages of 30–39 years. One third of the participants 33.8 % attended primary education, Orthodox Christianity accounts for 302 (76.7 %) and around half of the participants 48.4 % were Amhara by ethnicity. The median monthly income of the participants were 1000 birr and ranges from 100 to 9100 Ethiopian birr (Table 1).

**Table 1:** Distribution of HIV/AIDS patients by socio-demographic factors at ALERT hospital ART clinic in, 2015 (*n* = 417)

Variables	Categories	Frequency	Percent (%)
Sex	Male	166	39.8
	Female	251	60.2
Age	18–29	85	20.4
	30–39	157	37.7
	40–49	116	27.8
	≥50	59	14.1
Marital status	Single	130	31.2
	Divorced	69	16.5
	Widowed	69	16.5
	Married	149	35.7
Education	Unable to read and write	93	22.3
	Primary	141	33.8
	Secondary	137	32.9
	Above secondary	46	11
Religion	Orthodox	302	76.7
	Muslim	59	14.2
	Protestant	32	7.7
	Catholic	6	1.4
Ethnicity	Amhara	202	48.4
	Oromo	98	23.5
	Gurage	78	18.7
	Tigre	39	9.4
Occupation	governmental employee	73	17.5
	Private employee	112	26.9
	House wife	56	13.5
	Merchant	46	11
	Daily labor	46	11
	Student	84	20.1
Income	<1260	244	58.5
	≥1260	173	41.5

### Clinical and psychosocial characteristics of the respondents

Regarding clinical characteristics of study participants, 390 (93.5 %) had known their HIV sero positive status greater than for 12 months before the time of data collection. Around three fourth (72.4 %) of respondents were with CD4 count less than or equal to 500cells/μL. Half of study participants 48.2 % had WHO clinical stage I. From all study participants, 256 (61.4 %) had poor social support, 254 (58 %) had perceived HIV stigma and half of the respondents 50.1 % had poor medication adherence. From all study participants 50 (12 %) had co-morbid TB and100 (24 %) were currently substance (khat, cigarette and alcohol) users (Table 2).

**Table 2:** Distribution of clinical, psychosocial, medication adherence and substance use factors among patients with PLWHA at ALERT hospital ART clinic in, 2015 (*n* = 417)

Variables	Categories	Frequency	Percent (%)
Duration to know HIV status	≤12 months	27	6.5
	>12 months	390	93.5
CD4 count	≤500	302	72.4
	>500	115	27.6
Stage of HIV/AIDS	I	201	48.2
	II	121	29
	III	77	18.5
	IV	18	4.3
Comorbid disease	TB	50	12
	Others	45	10.8
	No	322	77.2
Good social support	Good	161	38.6
	Poor	256	61.4
Perceived HIV stigma	Yes	242	58
	No	175	42
Medication adherence	Good	204	49.9
	Poor	213	50.1
Substance ever use	No	142	34
	Yes	275	66
Substance current use	No	317	76
	Yes	100	24

### Prevalence of depression and anxiety among HIV/AIDS patients

The prevalence of co-morbid depression and anxiety among HIV patients in this study was 24.5 %. The prevalence of depression and anxiety among HIV patients in this study was 41.2 % (172) and 32.4 % (135) respectively.

### Factors associated with depression & anxiety among patients with HIV/AIDS

Multivariate logistic regression analysis revealed that having HIV perceived stigma, poor medication adherence, HIV stage III and poor social support were significantly associated with depression. Being female, being divorced and having HIV perceived stigma and co morbid TB illness were significantly associated with anxiety.

By using multivariate logistic regression individuals having poor social support (AOR = 2.02, 95 % CI (1.25, 3.27)) were associated with depression, those who had poor social support 2times more likely to develop depression than those who had good social support. Having HIV stage III (AOR = 2.80, 95 % CI (1.50, 5.21) were associated with depression. being on HIV stage III was2.8times more likely to exposed depressions compared to that of HIV stage I.

Similarly, having HIV perceived stigma had 3.6 times more likely to have depression as compared to patients who did not report perceived stigma (AOR = 3.60, 95 % CI (2.23, 5.80)). Patients who had poor medication adherence (AOR = 1.61, 95 % CI (1.02, 2.55) were associated with depression. Those with poor medication adherence were 1.6times more likely to develop depression as compared to that of good medication adherence (Table 3).

**Table 3:** Factors associated with depression among HIV patients at ALERT hospital ART clinic in, 2015 (*n* = 417)

Explanatory variables	Depression		COR, 95 % (CI)	AOR, 95 % (CI)
	Yes	No		
Age				
18–29	48	37	2.03 (1.03,3.99)	1.91 (.87,4.18)
30–39	58	99	0.92 (0.49,1.69)	1.02 (0.50,2.08)
40–49	43	73	0.92 (0.48,1.76)	1.20 (0.58,2.52)
≥ 50	23	36	1	1
Educational status				
Unable to read and write	50	37	2.33 (1.33,4.78)	2.05 (0.83,5.09)
Primary education	57	85	1.15 (0.59,2.26)	1.00 (0.44,2.23)
Secondary education	47	92	0.88 (0.45,1.73)	0.94 (0.43,2.06)
Diploma and above	18	31	1	1
Stage of HIV				
Stage one	64	137	1	1
Stage two	47	74	1.36 (0.85,2.18)	1.66 (0.98,2.83)
Stage three	49	28	3.75 (2.16,6.50)	2.80 (1.50,5.21)*
Stage four	12	6	4.28 (1.54,11.92)	3.03 (0.92,9.98)
CD4 cell count				
< =500cell/μL	137	165	1.90 (1.20,3.00)	1.39 (0.82,2.37)
> 500cell/μL	35	80	1	1
Perceived stigma				
Yes	130	112	3.68 (2.39,5.65)	3.60 (2.23,5.80)**
No	42	133	1	1
Medication adherence				
Poor	106	107	2.07 (1.39,3.08)	1.61 (1.02,2.55)*
Good	66	138	1	1
Social support				
Good	44	117	1	1
Poor	128	128	2.66 (1.74,4.06)	2.02 (1.25,3.27)*

*P*-value for hosmer and lemeshew test = 0.402 depression

Others comorbid illness = diabetes mellitus, hypothyroidism, epilepsy and hypertension

Significant association (* = *p*-value <0.05 and ** = *p*-value < 0.01), *n* = sample size

During multivariate logistic regression being female were 3 times (AOR = 3.13, 95 % CI (1.80, 5.44)) more likely to develop anxiety compared to male and being divorced were 2.5times (AOR = 2.51, 95 % CI (1.26, 5.00)) more likely to develop anxiety compared with married. Among clinical factors co morbid TB illness had 2.7times (AOR = 2.74, 95 % CI (1.37, 5.47)) more likely to develop anxiety as compared to that of no co morbid illness. Additionally, being stigmatized (AOR = 4.00, 95 % CI (2.40, 6.69)) were associated with anxiety. Those with stigmatized had 4times more likely develop anxiety as compared with not stigmatized (Table 4).

**Table 4:** Factors associated with anxiety among HIV patients at ALERT hospital ART clinic, 2015 (*n* = 417)

Explanatory variables	Anxiety		COR, 95 % (CI)	AOR, 95 % (CI)
	Yes	No		
Sex				
Male	30	136	1	1
Female	105	146	3.26 (2.04,5.21)	3.13 (1.80,5.44)**
Marital status				
Single	39	91	1.25 (0.74,2.12)	1.14 (0.62,2.10)
Divorced	32	37	2.53 (1.39,4.60)	2.51 (1.26,5.00)*
Widowed	26	43	1.77 (0.96,3.25)	1.31 (0.65,2.61)
Married	38	111	1	
Occupational status				
Student	34	40	2.51 (1.28,4.92)	1.89 (0.84,4.24)
Daily labor	17	29	1.73 (0.80, (3.76)	1.37 (0.56,3.35)
Merchant	10	36	0.82 (0.35,1.93)	1.08 (0.41,2.90)
House wife	18	38	1.40 (0.66, 2.95)	0.78 (0.32,1.89)
Private employee	35	77	1.34 (0.71,2.53)	1.26 (0.60,2.62)
Government employee	21	62	1	1
Comorbid disease with HIV				
TB	24	26	2.21 (1.21,4.01)	2.74 (1.37,5.47)*
Others	16	29	1.32 (0.68,2.54)	1.38 (0.66,2.88)
No disease	95	227	1	
Perceived stigma				
Yes	105	137	3.70 (2.32,5.92)	4.00 (2.40,6.69)
No	30	145	1	**
Good social support				
Yes	48	134	1	1
No	87	148	1.64 (1.01,2.50)	1.25 (0.76,2.05)

*P*-value for hosmer and lemeshew test = 0.540

Others co morbid illness = diabetes mellitus, hypothyroidism, epilepsy and hypertension

Significant association (* = *p*-value <0.05 and ** = *p*-value < 0.01)

## Discussion

This study revealed that the prevalence of co-morbid depression and anxiety was 24.5 %. Specifically, 41.2 % had depression and 32.4 % had anxiety. Perceived HIV stigma, poor social support, HIV stage III and poor medication adherence were significantly associated with depression. Being female, being divorced, having co morbid TB illness and perceived HIV stigma were also significantly associated with anxiety.

The prevalence of depression in the present study was 41.2 %. Regarding to prevalence, the current study result is line with other studies carried out in two areas of Ethiopia, USA and Denmark, in which the prevalence estimates were reported to be 38.94, 43.9, 44, 38 % respectively [20–23].

On the other hand, the present study findings was lower than the studies done in Albanian, China, India and Cameron in which the prevalence's were reported to be 58.75, 73.1, 66.3 and 63 % respectively [18, 24–26]. The variation of the prevalence might be due to different sample size used and different instruments used to assess depression. Only 100 participants were included in Cameron and PHQ-9 with the cutoff point greater than or equal to 9 [26], 320 participants in china and using CES-D with the cutoff points greater than or equal to16 [18], in India 160participants and using CES-D [24] and about 79 participants in Albania [25].

The result of the study was higher than the studies conducted in south Africa, Ghana, Nigeria, USA, Brazil, India, in which the prevalence were reported to be 25.4, 5.4, 23.1, 32, 14.15 and 35.5 % respectively [16–22, 24, 26–42]. The use different measurement tools used to assess depression and sample size difference that were in Ghana using DASS greater than or equal to 10 and 107 participants [37], in Nigeria 130 participants and using clinical interview DSM-IV [19] and in India using BDI-II and 152 participants [27].

Regarding to associated factors, having HIV stage III was associated with depression, which was 2.8 times more likely to develop depression as compared to that of HIV stage one patients. This was supported with the study done at Debirebirhan hospital in Ethiopia [21].

Perceived HIV stigma was strongly associated with depression in this study, which was 3.6 times having depression as compared with non-stigmatized HIV patients, which was supported with the studies done in Ethiopia at Debirbirahan hospital and USA [21, 35]. Stigma increase levels of fatigue and decrease attention or felling of worthlessness [35].

Having poor social support was 2 times more likely to develop depression as compared with those who had good social support that was comparable with the studies done in Ghana, India and two areas in china [17, 18, 43, 19]. HIV/AIDS patients preferred to avoid seeking help from others and avoid opening up about their health due to social stigma towards themselves, which increases their isolation and loneness [18, 19].

Having poor medication adherence was 1.6 times more likely to develop depression compared with those who had good medication adherence. This was supported by the study done in USA that was perceived HIV stigma and depression were the two interconnected factors that were aggravated poor medication adherence [35].

The prevalence of anxiety in this study was 32.4 %, which was similar to the studies done in USA, South Africa, Canada and western Europe together, in which the prevalence were reported to be 33, 30.6, 33.4, 33.3 % respectively [16, 27, 37, 44].

On the other hand, the current study findings was lower than the studies done in Albanian and two areas of china, in which the prevalence were reported to be 82.3, 45.6, 49 % respectively [18, 25, 43]. The possible reason might be the difference of tools used to assess anxiety and different sample size used. Which was 320 and 800 participants in two areas of China respectively and the same measurement tool used to assess anxiety, which was Zung self-rating anxiety scale with the cut of points greater than or equal to 50 and greater than or equal to 40 respectively.

Additionally, our findings was higher than the studies done in Ethiopia at Debrebirhan hospital, Ghana, Thailand, Brazil and Asia, in which the prevalence were reported to be 22.2, 7.2, 16.3, 12.6, 20.3 % respectively [30, 37, 45, 46, 19]. The above prevalence variations might be due to the difference in sample size used and different measurement tools used to assess anxiety. In Ethiopia and Brazil studies done to assessed the prevalence of anxiety by using BAI greater than or equal to 22 and 436 and 346 of their study participants respectively. In Thailand to assessed anxiety symptoms by using HADS with the cut of points greater than or equal to 11 and 251 participants and in Ghana used to assess anxiety by using DASS with the cut of points greater than or equal to 8 and 107 participants.

Being female had 3 times to develop anxiety as compared to male that was similar to the studies done in Ethiopia and Ghana [30, 35]. Being female is the exposure of acute life experience, low social interaction and had less social support from friends and families might be increased risk of anxiety [30, 35].

The present study revealed that being divorced was significantly associated with anxiety. In this study, being divorce was 2.5 times risk of developing anxiety as compared to married. This study was supported study done at Debretabor hospital in Ethiopia that was one of the outcome of divorce was affects the whole family structure and which leads to low self-confidence and financial problem [30].

Having TB/HIV co-infection had 2.7 times risk of developing anxiety as compared with those who had no co morbid illness. The possible reason might be drug-drug interaction, increase adverse effects and co-infection leads to high level of stigma and discrimination by verbal, gossip and name calling may lead to high rate of anxiety.

HIV perceived stigma had 4 times risk of developing anxiety as compared with those who had non-stigmatized HIV patients. This outcome was consistent by the studies done in Ethiopia and South Africa that were stigma was associated with anxiety [27, 30].

## Conclusion

The prevalence of co-morbid depression and anxiety (24.6 %), and prevalence of depression (41.2 %) and anxiety (32.4 %) was high. Having perceived HIV stigma, HIV Stage III, poor social support and poor medication adherence were significantly associated with depression. Whereas being female, being divorced, having co morbid TB and perceived HIV stigma were associated with anxiety. Ministry of Health should develop guidelines to screen and treat depression and anxiety among TB patients. Further research on risk factors of depression and anxiety should be conducted to strengthen and broaden these findings.
